# Local control of superconductivity in a NbSe_2_/CrSBr van der Waals heterostructure

**DOI:** 10.1038/s41467-023-43111-7

**Published:** 2023-11-09

**Authors:** Junhyeon Jo, Yuan Peisen, Haozhe Yang, Samuel Mañas-Valero, José J. Baldoví, Yao Lu, Eugenio Coronado, Fèlix Casanova, F. Sebastian Bergeret, Marco Gobbi, Luis E. Hueso

**Affiliations:** 1 CIC nanoGUNE BRTA, Donostia-San Sebastian, Spain; 2 Instituto de Ciencia Molecular (ICMol), Universitat de València, Paterna, Spain; 3 Centro de Física de Materiales (CFM-MPC) Centro Mixto CSIC-UPV/EHU, Donostia-San Sebastian, Spain; 4 IKERBASQUE, Basque Foundation for Science, Bilbao, Spain; 5 Donostia International Physics Center (DIPC), E-20018 Donostia–San Sebastián, Spain

**Keywords:** Superconducting properties and materials, Superconducting devices, Two-dimensional materials

## Abstract

Two-dimensional magnets and superconductors are emerging as tunable building-blocks for quantum computing and superconducting spintronic devices, and have been used to fabricate all two-dimensional versions of traditional devices, such as Josephson junctions. However, novel devices enabled by unique features of two-dimensional materials have not yet been demonstrated. Here, we present NbSe_2_/CrSBr van der Waals superconducting spin valves that exhibit infinite magnetoresistance and nonreciprocal charge transport. These responses arise from a unique metamagnetic transition in CrSBr, which controls the presence of localized stray fields suitably oriented to suppress the NbSe_2_ superconductivity in nanoscale regions and to break time reversal symmetry. Moreover, by integrating different CrSBr crystals in a lateral heterostructure, we demonstrate a superconductive spin valve characterized by multiple stable resistance states. Our results show how the unique physical properties of layered materials enable the realization of high-performance quantum devices based on novel working principles.

## Abstract (web summary)

Two-dimensional magnets and superconductors are emerging as tunable building blocks for quantum computing and superconducting spintronic devices. Here, Jo et al. demonstrate NbSe_2_/CrSBr van der Waals superconducting spin valves that exhibit infinite magnetoresistance and nonreciprocal charge transport, arising from a unique metamagnetic transition in CrSBr.

## Introduction

Heterostructures formed by ferromagnetic and superconducting materials have recently attracted enormous interest, as they host intriguing quantum phenomena such as spin-triplet or topological superconductivity that may provide novel solutions for spintronics and quantum computing^1–9^. In particular, superconducting spintronics makes use of the interplay between dissipationless supercurrents and spin-polarized quasiparticles emerging at superconductor/ferromagnet interfaces to achieve long-range spin transport and high-performance spintronic devices^10–13^. An important finding in this field is the discovery of infinite magnetoresistance (MR) in the so-called superconducting spin valve, a large-area device (typically a few mm^2^) composed of a thin superconducting layer sandwiched between two insulating ferromagnets^14–17^.

The recent discovery of two-dimensional (2D) superconductors and magnets has enabled the fabrication of van der Waals (vdW) heterostructures showing great potential for superconducting spintronics^18–25^. Devices based on these systems benefit from the atomically sharp interfaces characteristic of vdW heterostructures, but so far they have not exploited other remarkable properties of vdW materials, such as the magnetic field anisotropy in 2D superconductors^26–29^ or the peculiar magnetic configuration in some vdW magnets^30–35^.

Here, we demonstrate different nanoscale van der Waals superconducting spin valve architectures that exhibit infinite MR, multistep resistance switching, and nonreciprocal charge transport. Unlike conventional superconducting spin valves based on a three-layer system, our devices consist of a simpler bilayer composed of a vdW superconductor NbSe_2_ and a 2D magnet CrSBr. By exploiting a magnetic field-induced transition in CrSBr from a state with zero magnetization to a state with finite magnetization, we can achieve controllable and reversible suppression of the superconductivity of NbSe_2_ in a spatially confined region approximately 20 nm wide. This results in a sharp switching in the resistance from zero to a finite value, yielding an infinite MR reminiscent of conventional three-dimensional (3D) superconducting spin valves. Moreover, by integrating different CrSBr crystals in a lateral heterostructure, we demonstrate an unprecedented superconducting spin valve characterized by multiple resistance states that can be selected by applying different magnetic fields. Our results show how the distinctive properties of 2D materials offer the possibility to engineer superconducting devices with performances comparable to those of their 3D counterparts, yet miniaturized and based on different physical mechanisms.

**Fig. 1:**
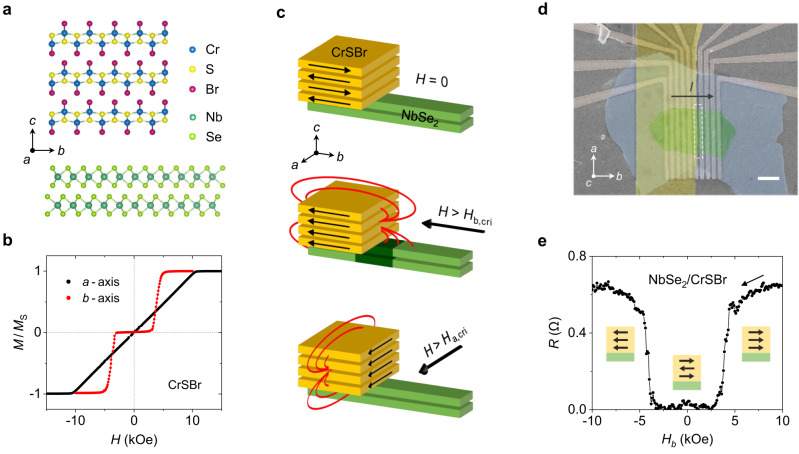
Superconducting switching in a NbSe_2_/CrSBr heterostructure. **a** Crystal structure of CrSBr and NbSe_2_ (side view). **b** Magnetization of a CrSBr crystal at 2 K. A parallel state in CrSBr is reached through the application of an in-plane magnetic field of *H* = 10 kOe and 3 kOe for the *a*-axis and *b*-axis, respectively. The magnetization is normalized to its saturated value in the parallel state. **c** Illustration of a stray field effect for the different magnetic states of CrSBr. When CrSBr (yellow) is in the parallel state saturated along its *b*-axis (middle panel), the out-of-plane component of a magnetic field penetrates the NbSe_2_ channel (green) in the region near the CrSBr flake’s edge (dark green region). **d** Colored scanning electron microscope image of a NbSe_2_/CrSBr device. The NbSe_2_/CrSBr heterostructure is placed on prepatterned parallel electrodes used to probe a resistance in different regions of the NbSe_2_ channel. NbSe_2_, CrSBr, and hBN flakes are colored green, yellow, and blue, respectively. The dashed white rectangle indicates the edge region. The scale bar represents 2 μm. **e** Superconducting magnetoresistance measured in NbSe_2_(5 ML)/CrSBr (Device 1) in the region close to the edge of the CrSBr flake by applying a magnetic field along the crystallographic *b*-axis of CrSBr, at 5 K. A sharp transition between the superconducting and the normal state occurs as the CrSBr is switched from its antiparallel to its parallel state.

The crystal structure of CrSBr is shown in Fig. 1a. Bulk CrSBr is a layered metamagnet in which the magnetic coupling is ferromagnetic within each layer, and antiferromagnetic between adjacent layers^36–41^. Interestingly, the magnetization of the layers can be switched from antiparallel (AP) to parallel (P) by the application of an external magnetic field^36^. In particular, when a magnetic field (*H*) is applied in a direction parallel to the magnetic easy axis of CrSBr (the crystallographic *b*-axis), a sharp AP-to-P transition is recorded at *H*_b_ = 3 kOe (Fig. 1b). Conversely, a smooth AP-to-P transition is recorded when applying a magnetic field parallel to the in-plane hard axis (*a*-axis), reaching a saturated P state at *H*_a_ = 10 kOe.

Figure 1c schematically shows the working principle of our superconducting spin valve. A few-layer-thick elongated NbSe_2_ flake is selected as a charge transporting channel. A wider CrSBr flake is placed on the NbSe_2_ channel, in such a way that its *b*-axis is aligned parallel to the NbSe_2_ flake’s long edge and it covers only a part of the NbSe_2_ flake. An in-plane magnetic field triggers the AP-to-P transition in CrSBr, which acquires net magnetization and generates out-of-plane stray fields that are localized at the edges of the CrSBr flake. Due to our device geometry, the stray fields penetrate the NbSe_2_ channel when a magnetic field is applied along the *b*-axis. Since a superconducting state in NbSe_2_ is much more sensitive to out-of-plane than in-plane magnetic fields, the externally applied in-plane field does not significantly affect the superconductivity^27^, while the out-of-plane stray fields efficiently suppress it. This causes a sudden change in the resistance state from zero to a finite value, yielding a sharp superconducting MR response. This effect only occurs in the region of a NbSe_2_ close to the CrSBr flake edge, where the stray fields are localized (see Fig. 1c). It is important to note that if a magnetic field is applied along the *a*-axis, the CrSBr also acquires net magnetization, but it generates out-of-plane stray fields far from the NbSe_2_ channel in our heterostructure, not affecting the superconducting state of the NbSe_2_ (bottom image in Fig. 1c).

Figure 1d shows a scanning electron microscope image of a representative NbSe_2_/CrSBr device. The NbSe_2_/CrSBr/hBN heterostructure was fabricated using a van der Waals pick-up technique and transferred onto a set of prepatterned parallel electrodes (see Methods). This device geometry allows us to record the resistance of the NbSe_2_ channel and its dependence on a magnetic field in three different regions: (i) in a pristine NbSe_2_, far from the CrSBr, (ii) near the edge of the CrSBr flake (edge region), and (iii) in the NbSe_2_ fully covered by the CrSBr. To perform these measurements, two outer electrodes are used to apply a bias current, while a voltage drop is measured across different pairs of electrodes by applying an in-plane magnetic field parallel to either the *b*- or *a*-axis of CrSBr.

We initially focus on the behavior of NbSe_2_ in the edge region. Figure 1e displays the MR recorded in an NbSe_2_(5 ML)/CrSBr heterostructure (Device 1) when applying a magnetic field along the CrSBr’s *b*-axis. A sharp resistance increase is observed at the magnetic transition field of CrSBr, indicating that in this region the superconductivity of NbSe_2_ is suppressed. This superconducting switching shows infinite MR, defined as usual as MR (%) = (*R*_max_ − *R*_min_)/ *R*_min_ × 100^15^.

**Fig. 2:**
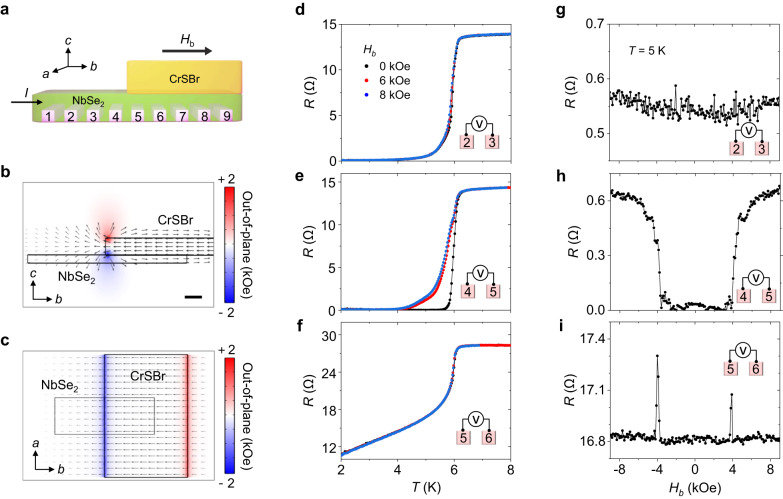
Position-dependent characterization of superconducting magnetoresistance in NbSe_2_(5 ML)/CrSBr (Device 1). **a** Schematic image of our NbSe_2_/CrSBr device. Numbers indicate electrode positions. The edge of the CrSBr flake is placed between electrodes 4 and 5. **b**, **c** Side (**b**) and top (**c**) view of the simulated map for the stray field’s out-of-plane component. When an external magnetic field *H* is applied along the *b*-axis, an out-of-plane stray field is generated at the edge of the CrSBr flake. The scale bar indicates 20 nm. **d**–**f** Temperature-dependent resistance measured at different positions in *H*_b_ = 0, 6, and 8 kOe. There is a negligible change upon applied magnetic fields in a pristine NbSe_2_. Superconductivity switching in the NbSe_2_ occurs at the edge of the CrSBr flake, whereas a non-zero resistance state is recorded for the NbSe_2_ under the CrSBr flake. **g**–**i** Magnetic field dependence of the resistance measured at different positions. Sharp superconducting magnetoresistance is observed at the edge of the CrSBr flake due to the stray out-of-plane field, and the effect decays as the measured position moves away from the CrSBr edge.

Figure 2 shows a comprehensive analysis of the NbSe_2_/CrSBr (Device 1) measuring at different positions. Multiple contact electrodes are employed, while the width of both electrodes and channels is 300 nm (Fig. 2a). This position-dependent measurement allows us to disentangle the stray field effect from other possible contributions, such as the interfacial inverse proximity effect between NbSe_2_ and CrSBr. We first confirm the cross-sectional map of a stray field distribution by a COMSOL Multiphysics software; it indicates that the stray field is maximum at the edge of the CrSBr (Fig. 2b). The CrSBr is considered to be in the P state with a thickness of 20 nm and saturated along the *b*-axis. The calculated full width at half maximum of the stray field is 20 nm and its maximum amplitude reaches 1.3 kOe (see Supplementary Fig. 1). We note that an out-of-plane magnetic field of this magnitude is sufficient to suppress the superconductivity of NbSe_2_^26–29^. Moreover, our simulation confirms that when the CrSBr is magnetized along the *b*-axis, the out-of-plane component of a stray field is localized at the flake’s edges, bordering the NbSe_2_ channel (see Fig. 2b, c). On the other hand, when the CrSBr is magnetized along the *a*-axis, the out-of-plane component of a stray field does not reach the NbSe_2_ flake, explaining the absence of superconducting MR for this magnetic field orientation (Supplementary Fig. 2).

Figure 2d–i shows the temperature dependence of resistance, *R*(*T*), and its magnetic field dependence, *R*(*H*), measured at 5 K in three different regions in Device 1, which are: (i) pristine NbSe_2,_ (ii) NbSe_2_ partially covered by CrSBr (edge region), (iii) NbSe_2_ fully covered by CrSBr.

In the pristine NbSe_2_ (region i) (Fig. 2d), there is a negligible difference in the *R*(*T*) curves measured from *H*_b_ = 0 to 8 kOe. Correspondingly, the MR recorded at 5 K is flat (Fig. 2g). Larger in-plane fields are required to significantly affect the superconductivity in NbSe_2_.

In contrast, a clear change in the *R*(*T*) curves can be observed in the edge region (ii) (Fig. 2e). The NbSe_2_ exhibits two different states depending on the magnitude of the applied magnetic field. In particular, a detected resistance near 5 K is zero and finite for *H*_b_ = 0 Oe and *H*_b_ = 6 kOe, respectively. This behavior can also be observed in the *R(H)* curve recorded at 5 K, which shows a sharp switching of resistance from zero to a finite value near 4 kOe when sweeping a magnetic field along the *b*-axis, corresponding to infinite MR (Fig. 2h). On the other hand, the MR recorded at the same device region with the application of a magnetic field along the CrSBr’s *a*-axis does not show a sharp jump in the resistance of NbSe_2_ (Supplementary Fig. 3). This indicates that the superconducting MR is indeed caused by the localized stray fields emanating from the CrSBr in its P state, which only affect the superconductivity of the NbSe_2_ when the magnetization of the CrSBr is aligned along the *b-*axis, as sketched in Fig. 1c. Thus, the NbSe_2_ close to the CrSBr edge exhibits two stable resistance states, which can be controlled by a magnetic field. This behavior is similar to that of 3D superconducting spin valves, where the bistability results from the different relative alignments (parallel or antiparallel) of the magnetization of two ferromagnetic thin films. In our case, the two different resistance states are caused by the different alignments of the atomically thin magnetic sheets of a CrSBr flake.

Finally, in the region of NbSe_2_/CrSBr (iii), far from the CrSBr flake’s edge, the superconductivity of the NbSe_2_ is suppressed to a remarkable degree at low temperatures (Fig. 2f). This can be attributed to the inverse proximity effect at the NbSe_2_/CrSBr interface or to the strain-introduced in NbSe_2_ during the fabrication of a vdW heterostructure^42,43^. The interfacial origin of this behavior is supported by the observation that superconductivity suppression is more significant for thinner NbSe_2_ flakes. Indeed, a zero resistance state is not observed in a 5-ML-thick NbSe_2_ region below a CrSBr flake (Fig. 2f), whereas it is observed for an 11-ML-thick NbSe_2_ flake (Device 2, Supplementary Fig. 4). In any case, even though the superconductivity is suppressed due to this inverse proximity effect, a resistance drop is observed below 6 K regardless of applied magnetic fields (Fig. 2f). This is probably due to the fact that Cooper pairs leak from the uncovered (superconducting) NbSe_2_ to the adjacent CrSBr-covered (non-superconducting) NbSe_2_ within a Cooper pair penetration length. The MR measured in the same region (iii) is also flat (Fig. 2i), apart from narrow features appearing at *H*_b_ = ±4 kOe, corresponding to the switching field in the CrSBr. These features may be caused by stray fields generated by the presence of magnetic domain walls at the edge channel region during the magnetic transition.

Overall, the fact that sharp and infinite superconducting MR is observed only in the region near the edge of the CrSBr flake provides clear evidence that it originates from stray fields localized in that region. Therefore, our device engineering strategy enables the switching of a highly localized vertical magnetic field using an in-plane magnetic field.

**Fig. 3:**
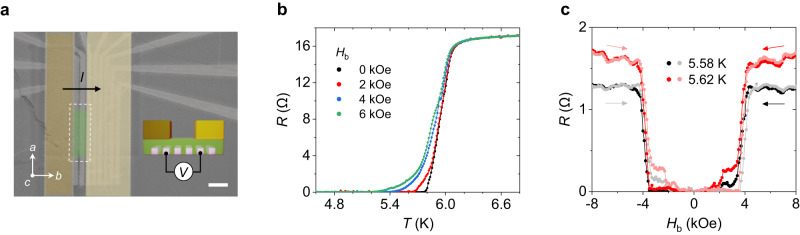
Multistate superconducting spin valve in NbSe_2_(4 ML) with two CrSBr flakes (Device 3). **a** Colored scanning electron microscope image of the measured device. The heterostructure composed of a NbSe_2_ flake (green) and two CrSBr flakes (dark and light yellow) on top of the NbSe_2_ is placed on prepatterned parallel electrodes. The scale bar represents 2 μm. A voltage is measured at the region (white dot box) which contains two CrSBr edges while applying a current through the *b*-axis. The schematic inset displays where the voltage is measured. **b** Temperature-dependence of the resistance upon different magnetic fields along the *b*-axis of CrSBr. Multiple resistance states are observed at the different fields in the temperature range between 5.5 K and 5.7 K. **c** Superconducting magnetoresistance on different temperatures. Multiple resistance states are observed at approximate magnetic field ranges of 0–2 kOe, 2–4 kOe, and above 4 kOe, which result from different switching fields of the two CrSBr flakes. Dark and light colors indicate the opposite sweep direction of a magnetic field at the same temperature. The asymmetric behavior might arise from multidomain magnetic structures in the thick CrSBr flake.

Our control of localized stray fields in a NbSe_2_/CrSBr heterostructure can be utilized to engineer a novel multistate superconducting spin valve. To this goal, we prepared a device (Device 3), consisting of a NbSe_2_ channel covered in different regions by two separated CrSBr flakes with different thicknesses and fully encapsulated by an hBN layer (Fig. 3a). We focus on the region containing the two CrSBr edges (see the inset), which corresponds to two single-edge superconducting spin valves connected in series. Figure 3b shows distinct *R*(*T*) traces recorded at different magnetic fields. Without a magnetic field, a sharp superconducting transition occurs at 5.93 K, indicating a superconducting behavior (black). When an external magnetic field *H*_b_ = 6 kOe is applied, the superconducting transition is suppressed and a zero resistance state is reached at a lower temperature, similar to what was observed in the device with a single CrSBr edge (see Fig. 2e for comparison). In contrast, an important difference in the *R*(*T*) is observed when a lower field is applied. While for a single-edge device, there are two resistance states (zero and finite), for the two-edge case, three different states are observed near 5.6 K (Fig. 3b). This behavior can be understood considering that the two CrSBr flakes with different thicknesses possess different AP-P switching fields, so they affect the NbSe_2_ resistance differently. As a result, in the temperature region between 5.5 K and 5.7 K, the device resistance can be switched among multiple states. This is further exemplified in Fig. 3c which shows the *R*(*H*) of the two-edge device. In this case, three resistance states, in the approximate field ranges of 0–2 kOe, 2–4 kOe, and above 4 kOe, are observed. We ascribe these regions to the stray fields introduced by the metamagnetic transitions of the two CSB flakes. Importantly, we note that the lowest resistance state is zero, so this device is also a multistate superconducting spin valve.

**Fig. 4:**
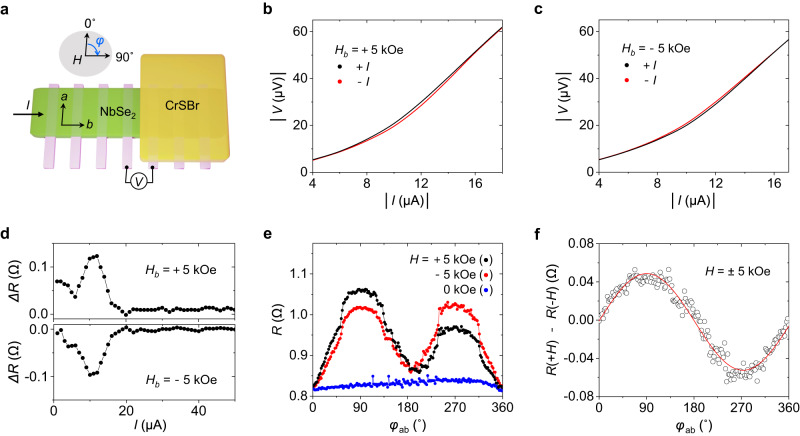
Nonreciprocal charge transport in NbSe_2_(10 ML)/CrSBr (Device 4). **a** Sketch of measurement configuration. All nonreciprocal responses were measured in the NbSe_2_ channel at the edge of the CrSBr flake through the bottom electrodes. **b**, **c** Voltage drop measured for different current polarities (red/black curves) by applying a magnetic field of +5 kOe (**b**) and −5 kOe (**c**) along the *b*-axis of CrSBr. **d** Resistance difference for different applied magnetic fields, collected from (**b**) and (**c**). The resistance difference is defined as Δ*R* = *R*( + *I*) − *R*(−*I*). The sign of Δ*R* is observed to change depending on the direction of a magnetic field, indicating nonreciprocal charge transport. **e** Angle-dependent resistance at a positive and negative magnetic field of 5 kOe and a current of +10 μA. *φ* represents the angle between the in-plane magnetic field *H* and the electrode, shown in (**a**). **f** Dependence of the difference in the resistance measured with opposite fields as a function of the angle *φ*. The difference is maximum/minimum at *φ* = 90° and 270°, respectively, i.e., when a magnetic field is applied along the CrSBr’s *b*-axis. All measurements were performed at 6.47 K.

In this final section, we show that the switchable stray fields emerging from a CrSBr flake in its P state can lead to another effect, namely an unconventional nonreciprocal charge transport (in Fig. 4). Such nonreciprocity is expected in noncentrosymmetric materials^44–46^ when time-reversal symmetry is broken by the application of a magnetic field. This nonreciprocal transport, also called unidirectional MR or magnetochiral anisotropy, is empirically described as *R* = *R*_0_ (1 + *γIB*), where *R*_0_ is the resistance at zero magnetic fields, *γ* is the coefficient of anisotropy, and *B* is the magnetic field^47,48^. In superconductors, this effect is enhanced in the resistive (quasi-particle) regime close to a critical current. This phenomenon has also been observed in flakes of layered superconductors such as NbSe_2_ and ion-gated MoS_2_, where a few-layer thickness effectively breaks the inversion symmetry. In these works, the nonreciprocal charge transport was recorded by applying a magnetic field in an out-of-plane direction through a whole flake region.

Here, we detected nonreciprocal transport in the NbSe_2_ region at the edge of a CrSBr flake when applying an in-plane magnetic field (Fig. 4a). Figure 4b shows a current-voltage (*I-V)* trace measured in the edge region of Device 4 at a fixed magnetic field of +5 kOe along the *b*-axis. To facilitate the comparison between positive and negative values, the absolute value of a current and voltage is plotted. The voltage measured at a positive current (black line) is higher than that measured at a negative current (red line). The situation is specular when the magnetic field direction is reversed (Fig. 4c). Figure 4d shows the difference in resistance for positive and negative currents (as obtained from Fig. 4b and 4c), defined as Δ*R* = *R*(+*I*) − *R*(−*I*). Δ*R* reaches a maximum at 10 μA and decays to zero at 20 μA, which is the critical current for the nonreciprocal transport in our system. The opposite sign of Δ*R* evidences the nonreciprocal response to an in-plane magnetic field.

Figure 4e displays the angular resistance measured at a constant current of +10 μA as a function of the orientation of an in-plane magnetic field. A unidirectional response to the magnetic field is observed when the field is aligned with the *b*-axes (90° and 270°), where the resistance difference measured at the opposite magnetic fields is as large as 5%. By contrast, no remarkable change is detected when the field is aligned with the *a*-axes (0° and 180°). The net difference between the resistance measured at +5 kOe and −5 kOe is plotted in Fig. 4f and displays the sinusoidal curve typical of nonreciprocal charge transport. An analogous behavior is also observed for a larger applied field (see Supplementary Fig. 5). This nonreciprocal response is only detected in the NbSe_2_ channel near the edge of the CrSBr flake. In this region, an in-plane external magnetic field introduces an effective stray field with an out-of-plane component, which causes the nonreciprocal effect, as previously reported for layered superconductors. The magnitude of the effect is quantified by the coefficient *γ*, which in our device reaches almost 4000 *T*^−1^*A*^−^^1^ for an in-plane field of 0.5 T, a value comparable to those previously measured in layered superconductors in the presence of an out-of-plane magnetic field^44,49^. Thus, our approach further provides remarkable performance of a rectification effect in a very localized working area (approximately 20 nm) that can prevent affecting a whole device by an external magnetic field and be a crucial element for nanodevice scaling.

In conclusion, we have demonstrated a controllable nanoscale complete switching of the superconductivity on NbSe_2_ due to the AP-to-P magnetic transition in CrSBr. This effect is analogous to that reported in large-area superconducting spin valves, but based on a different physical mechanism and on a simpler nanoscale device architecture. In addition, we have further proved the advanced capability of our localized stray field engineering for device application with the demonstration of a multistate superconducting spin valve and with the report of a nonlinear rectification effect in a very localized region of NbSe_2_. All these phenomena are enabled by properties unique to layered materials, namely (i) the peculiar magnetic configuration of CrSBr, which can be switched from an AP to a P state at a low magnetic field, and (ii) the anisotropic superconductivity of NbSe_2_, which is more sensitive to out-of-plane than in-plane fields.

Our results propose a novel approach to develop superconducting spintronic devices based on 2D materials. The NbSe_2_/CrSBr bilayer devices described in this work are characterized by a novel working mechanism, a high figure of merit, and an architecture simpler than that of conventional superconducting spin valves.

## Methods

### Device fabrication

Prepatterned electrodes were designed on Si/SiO_2_ substrates using electron beam lithography, and 12 nm of Pt was deposited as the electrode. The width and length of the electrodes were 300 nm and 10 μm respectively, and the channel gap between them was 300 nm. Bulk 2D crystals of NbSe_2_ and hBN were purchased from HQ graphene. CrSBr crystals were synthesized by chemical vapor transport using the direct reaction of components in a stoichiometric ratio, mixing chromium (99.99%, Alfa-Aesar), sulfur (99.99%, Sigma-Aldrich), and bromine (99.9%, Sigma-Aldrich) with a 3% in mass excess of bromine, which acts as a transport agent^41^. Targeted 2D flakes were mechanically exfoliated on a clean Si/SiO_2_ substrate and a NbSe_2_/CrSBr/hBN heterostructure was fabricated in an Ar-filled glovebox by a dry-transfer method using a polycaprolactone polymer^50^. The thickness of the 2D flakes was cross-checked by optical contrast and atomic force microscopy measurement. Optical contrast was used to determine flake thickness during the fabrication in a glovebox. In total, the main results from 4 devices were presented in the text: Device 1 (NbSe_2_ of 5 ML) in Figs. 1 and 2, Device 2 (NbSe_2_ of 11 ML) in Supplementary Fig. 4, Device 3 (NbSe_2_ of 4 ML with two CrSBr flakes) in Fig. 3, and Device 4 (NbSe_2_ of 10 ML) in Fig. 4.

### Electrical measurement

A fabricated device was mounted in the sample stage of a PPMS (Physical Property Measurement System, Quantum Design) with a horizontal rotator. A Keithley 6221 was used to apply a current and a Keithley 2182 nanovoltmeter detected the voltage. A current of 1 μΑ was typically used considering a critical current of NbSe_2_ and a heating effect. A magnetic field and temperature were swept at constant rates in all measurements: 100 Oe/s and 0.2 K/min, respectively.

### Supplementary information

Supplementary Information

Peer Review File

## Data Availability

All data are available in the main text and the Supplementary Information. Additional data related to the findings in this study can be requested from the authors.
