# Supplementary material for: Local control of superconductivity in a NbSe2/CrSBr van der Waals heterostructure
Source: Nat Commun. 2023 Nov 9;14:7253. doi: 10.1038/s41467-023-43111-7 (PMC10636142; doi:10.1038/s41467-023-43111-7)
Supplement: Supplementary file 1 — Supplementary Information [file 41467_2023_43111_MOESM1_ESM.pdf]

Supplementary Information

# Local control of superconductivity in a $NbSe_2$/CrSBr van der Waals heterostructure

Junhyeon Jo[1,*], Yuan Peisen[1], Haozhe Yang[1], Samuel Mañas-Valero[2], José J. Baldoví[2], Yao Lu[3], Eugenio Coronado[2], Fèlix Casanova[1,4], F. Sebastian Bergeret[3,5], Marco Gobbi[3,4,*], Luis E. Hueso[1,4,*]

[1]CIC nanoGUNE BRTA, Donostia-San Sebastian, Spain

[2]Instituto de Ciencia Molecular (ICMol), Universidad de València, Paterna, Spain

[3]Centro de Física de Materiales CSIC-UPV/EHU, Donostia-San Sebastian, Spain

[4]IKERBASQUE, Basque Foundation for Science, Bilbao, Spain

[5]Donostia International Physics Center (DIPC), E-20018 Donostia–San Sebastián, Spain

[*]Corresponding authors:

j.jo@nanogune.eu; marco_gobbi001@ehu.eus; l.hueso@nanogune.eu

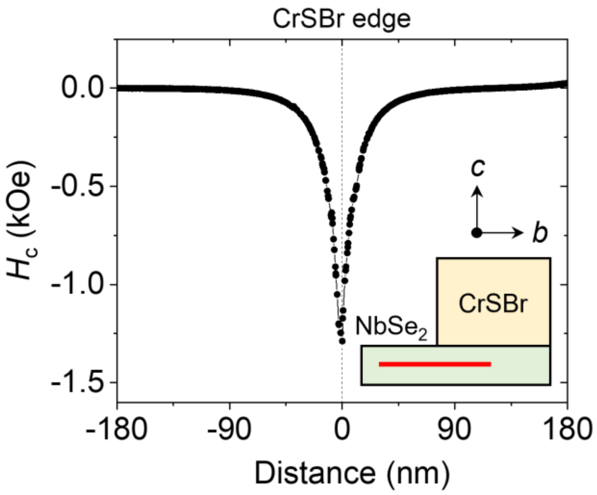


**Supplementary Fig. 1 | Out-of-plane stray field profile in a $NbSe_2$/CrSBr with an external magnetic field along the *b*-axis.** The out-of-plane component (*c*-axis) of a stray field is calculated along the red line. The stray field is maximum at the edge of the CrSBr flake. The full width at half maximum for the stray field is 20 nm.

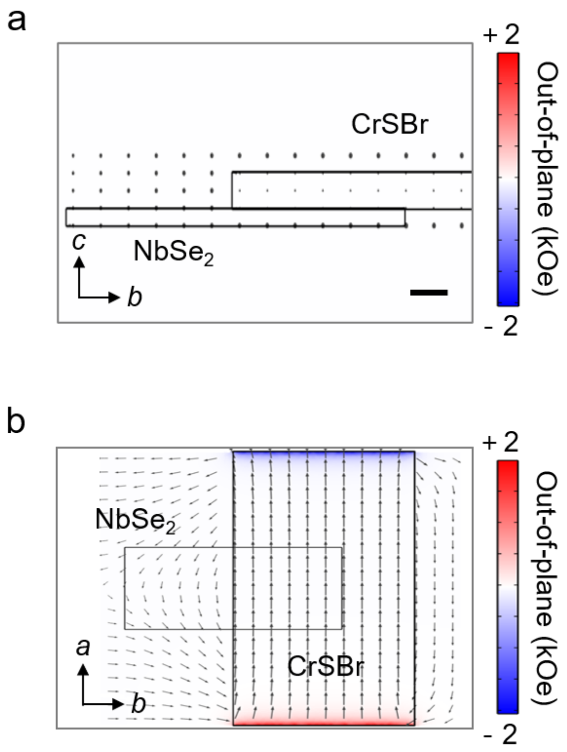


**Supplementary Fig. 2 | Stray field maps for an out-of-plane field component under an external magnetic field parallel to the *a*-axis. a,b** The side and top view of a stray field map. In this geometry, there is no out-of-plane component affecting the $NbSe_2$ channel. The scale bar indicates 20 nm.

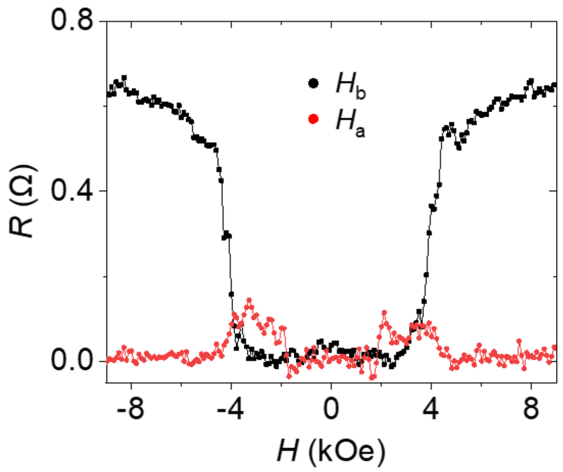


**Supplementary Fig. 3 | Superconducting magnetoresistance in a $NbSe_2$(5 ML)/CrSBr heterostructure (Device 1) varying on the direction of a magnetic field.** Magnetoresistance as a function of a magnetic field orientation. When a magnetic field is applied along the *b*-axis, a sharp magnetoresistive effect is observed (black curve). Applying the field along the *a*-axis does not produce the superconducting switching effect due to the absence of a stray field at the measured position (red curve). The device geometry is shown in Fig. 1.

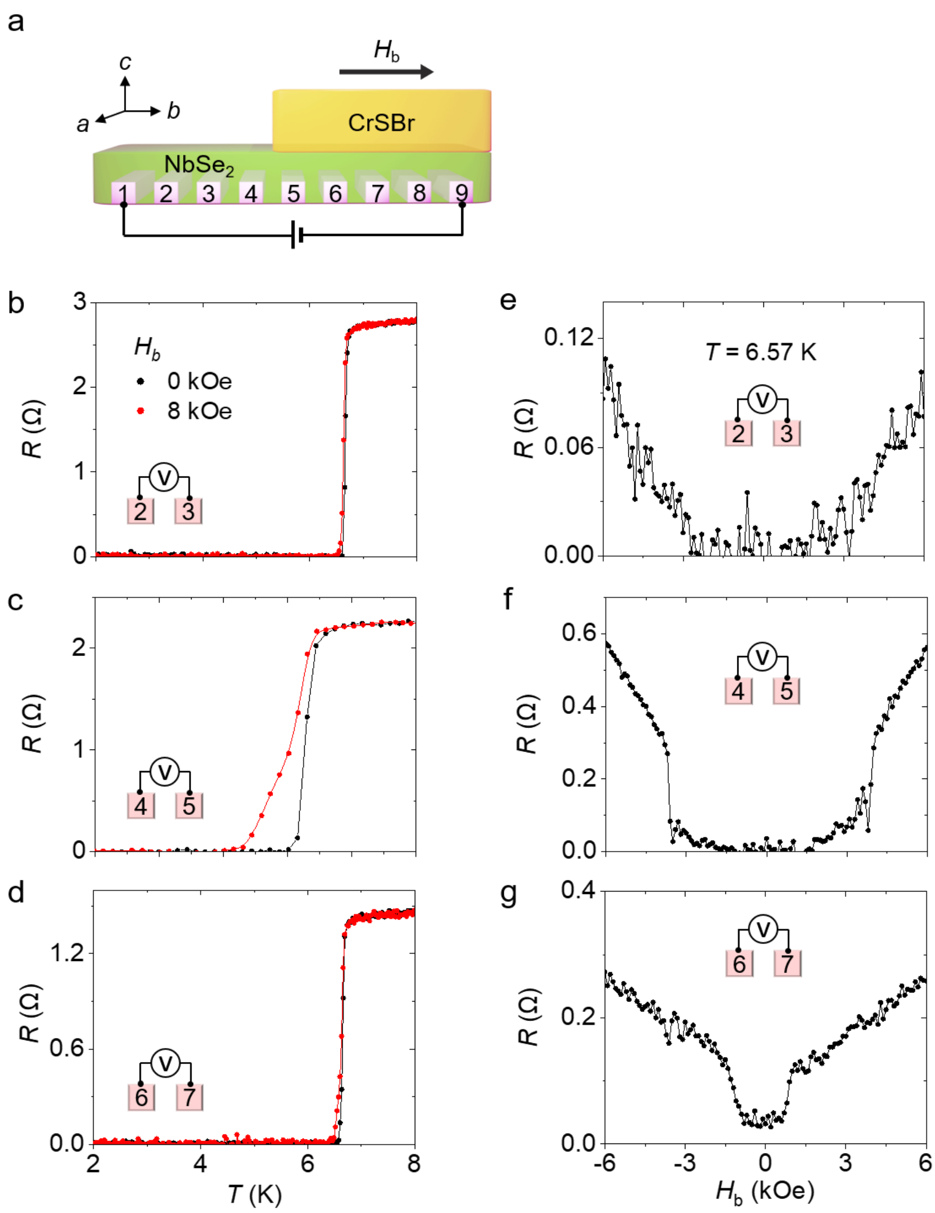


**Supplementary Fig. 4 | Position-dependent analysis in $NbSe_2$(11 ML)/CrSBr (Device 2). a** Schematic image of the $NbSe_2$/CrSBr device. Numbers indicate electrode positions. The edge of the CrSBr flake locates between electrode 4 and 5. **b-d** Temperature-dependent resistance at different positions at $H_b = 0$ kOe and $H_b = 8$ kOe. The superconducting switching behavior of the $NbSe_2$ only occurs at the edge of the CrSBr flake. A zero resistance state is observed in the region fully covered by CrSBr (compared with the $NbSe_2$(5 ML)/CrSBr in Fig. 2). **e-g** Magnetic field-dependent resistance at different positions. Shapr superconducting magnetoresistance is observed at the edge of the CrSBr flake.

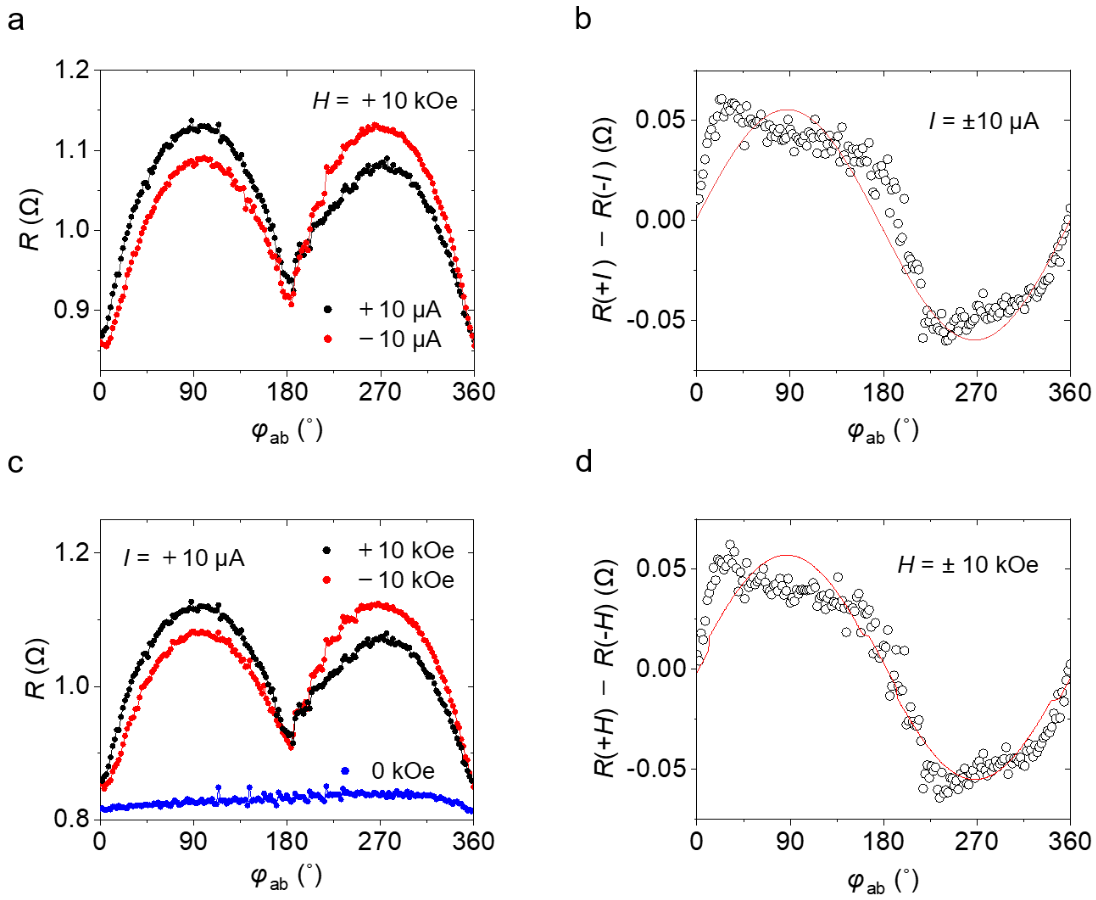


**Supplementary Fig. 5 | Angle-dependent nonreciprocal charge transport in $NbSe_2$(10 ML)/CrSBr (Device 4). a** Current-polarity dependence at a fixed magnetic field of +10 kOe. **b** Net resistance difference between a positive and negative current state. The obtained sinusoidal graph indicates the characteristic of nonreciprocal transport. **c** Magnetic field-polarity dependence with a fixed current of +10 μA. **d** Net resistance difference between a positive and negative magnetic field state. A sinusoidal graph is observed indicating nonreciprocal transport feature.
